# A Dorsal Epidural Herniated Disc Fragment Initially Presenting as Guillain-Barré Syndrome

**DOI:** 10.7759/cureus.25719

**Published:** 2022-06-07

**Authors:** Parth N Patel, Michael G Schloss, Kaveri Sharma, Poonam Dulai

**Affiliations:** 1 Physical Medicine and Rehabilitation, Mercy Hospital, Catholic Health, Rockville Centre, USA; 2 Neurology, Mercy Hospital, Catholic Health, Rockville Centre, USA

**Keywords:** guillain-barré syndrome (gbs), epidural, tumor excision with laminectomy, spine injury, disk fragment, dorsal spine, spine, spasticity, disk herniation

## Abstract

Guillain-Barré syndrome (GBS) is a rare autoimmune disorder that presents with neurological symptoms that can mimic other conditions. This mimicry can hide other important neurological diagnoses. Here, we present a rare case of thoracic myelopathy secondary to a sequestered dorsal epidural herniated disc fragment that initially presented with the classic findings of GBS.

A 58-year-old female presented with progressing bilateral lower extremity weakness, paresthesias, and absent bilateral lower extremity deep tendon reflexes. Lumbar magnetic resonance imaging (MRI) findings were disproportionate to presentation, and lumbar puncture fluid analysis revealed clear, colorless fluid with albuminocytological dissociation. The patient was diagnosed with GBS and treated with a short course of intravenous steroids followed by intravenous immunoglobulin. The patient later developed new-onset ulnar distribution paresthesias, lower extremity spasticity, constipation, and urinary retention that caused a decline in functional progress. Further investigation prompted evaluation with cervical and thoracic MRIs, which revealed a left dorsal epidural lesion at the T9-T10 level causing severe cord compression. The patient was definitively treated with a T9-T10 laminectomy and excision of the offending lesion. Pathology revealed collagenous tissue with fibroblastic proliferation, consistent with a sequestered fragment of the herniated intervertebral disc. The patient was further treated with both acute and subacute rehabilitation. She was eventually discharged home and was able to ambulate independently with a walker.

Dependency on positive albuminocytological dissociation in cases of potential GBS can lead to errors in diagnostic accuracy and delay appropriate treatment. Clinicians should remain mindful that GBS is a diagnosis of exclusion and MRI of the entire spine should be considered when the diagnosis of GBS is uncertain.

## Introduction

Guillain-Barré syndrome (GBS) is a rare autoimmune disorder of the peripheral nervous system which causes rapid symmetric ascending paralysis, often provoked by an infectious illness [1]. Occasionally, paralysis can progress to the diaphragm muscle and necessitate mechanical ventilation [1]. In addition, patients with acute GBS frequently present with decreased or absent deep tendon reflexes [1]. Although these classic findings are typically recognizable by clinicians, GBS has multiple variants and can present with vague symptoms of weakness, pain, and paresthesias, making it difficult to diagnose in its early stages due to its overlap with other conditions [2]. Here, we present a rare case of thoracic myelopathy secondary to a sequestered dorsal epidural herniated disc fragment that initially presented with the classic findings of GBS.

## Case presentation

A 58-year-old female with a medical history of hypertension, right hip slipped capital femoral epiphysis status post-surgical fixation with subsequent advanced hip and knee osteoarthritis, and obesity presented to the Emergency Department seeking evaluation for progressing bilateral lower extremity weakness and paresthesias over the course of several days. She denied any trauma or preceding illness. Prior to admission, she lived alone in a private home and ambulated with the help of a single point cane. Before admission, her medications included tramadol and gabapentin for pain control. There was no known history of osteopenia.

On the initial examination by the consulting neurologist, she exhibited profound bilateral lower extremity weakness. She was rated at 2/5 for flexion and extension strength at the knee and 1/5 plantarflexion and dorsiflexion strength at the ankle. In addition, her patellar and Achilles deep tendon reflexes were absent bilaterally, while her biceps and triceps reflexes were within normal limits at 2+. She did not exhibit any clonus or spasticity, and the Babinski sign was negative bilaterally. Sensation was decreased in her lower extremities bilaterally, but no clear neurologic level was documented. The remainder of the neurologic examination was unremarkable. Based on these findings, lumbar magnetic resonance imaging (MRI) and a lumbar puncture (LP) were ordered.

MRI of the lumbar spine demonstrated multiple mild disc herniations and mild canal stenosis at the L2-L3 interspace. Neurosurgery was consulted and deemed no intervention was necessary, stating that the patient’s symptoms were disproportionate to imaging results. LP fluid analysis revealed clear, colorless fluid with albuminocytological dissociation. Cerebrospinal fluid (CSF) protein was elevated at 113 mg/dL (reference range: 15-45 mg/dL) and white blood cell count was within normal limits at 5/µL (reference range: 0-5/µL). All other routine laboratory studies were within normal limits.

Considering the patient’s muscle weakness, absent deep tendon reflexes, and LP findings, the diagnosis of GBS was made. Treatment with a short course of dexamethasone was initiated, but did not lead to a significant improvement in symptoms. The patient received 10 mg of dexamethasone intravenously (IV) on day one, followed by 4 mg IV every six hours on day two. On day three, treatment was switched to intravenous immunoglobulin (IVIG) at a dosage of 400 mg/kg daily for five days, which led to functional improvement after completion of the course. Lower extremity muscle strength rating improved to 3/5 bilaterally throughout all muscle groups, and bilateral lower extremity reflexes returned to 2+. Despite this improvement, because the patient continued to require assistance with activities of functional daily living and ambulation, the decision was made to consult the physical medicine and rehabilitation service, who recommended admission to acute inpatient rehabilitation.

The patient’s acute rehabilitation course consisted of 90 minutes of physical therapy and 90 minutes of occupational therapy five days per week. On inpatient rehabilitation admission day four, she was noted to have increased muscle spasms. She was trialed on two muscle relaxants which provided some short-term relief. She first received 2 mg of tizanidine nightly and every eight hours as needed for four days but was then changed to 5 mg of baclofen every eight hours as needed for four days due to rising transaminases while using tizanidine. In addition, she continued to complain of right hip and knee pain with weight-bearing, which limited her ability to ambulate and participate in exercises. For these persistent complaints, she was started on 5 mg of oxycodone every eight hours as needed, which provided relief. By admission day seven, her left leg strength had improved to 4/5 throughout, but her right leg strength remained at 3/5 throughout. Two weeks into her acute inpatient rehabilitation stay, she progressed from requiring maximum assistance (greater than 75% of work being performed by the treating therapist) from two therapists for bed mobility and transfers to requiring moderate assistance (about 50% of work being performed by the treating therapist) for the same activities. She also improved from being non-ambulatory to being able to ambulate 25 feet using a self-propelling wheelchair under supervision by the treating therapist.

It was ultimately determined that the patient would require continued rehabilitation in a subacute facility. Prior to discharge to subacute rehabilitation, the patient developed new-onset bilateral ulnar distribution paresthesias, lower extremity spasticity, constipation, and urinary retention that caused a decline in functional progress. Diminishing functional improvements included a regression of functional status back to maximum assistance for transfers and decreased ambulatory distance. These findings prompted the clinician team to postpone discharge and obtain cervical and thoracic MRIs to evaluate for myelopathy.

**Figure 1:**
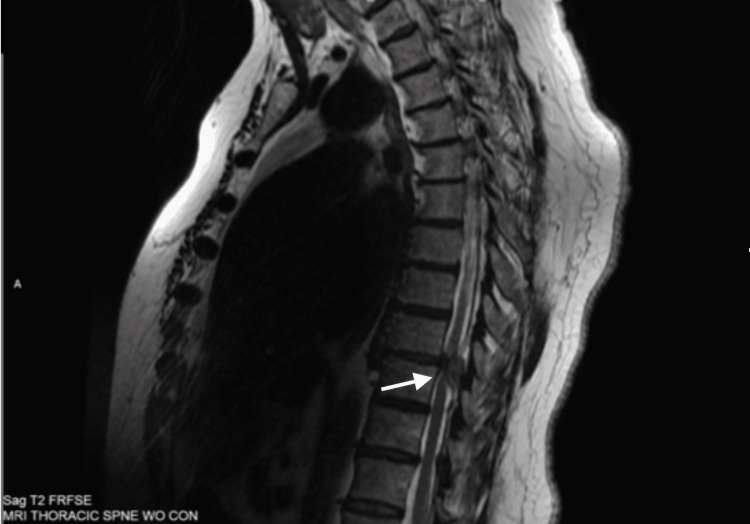
Sagittal view depicting dorsal epidural disc fragment centered at the T9-T10 level.

**Figure 2:**
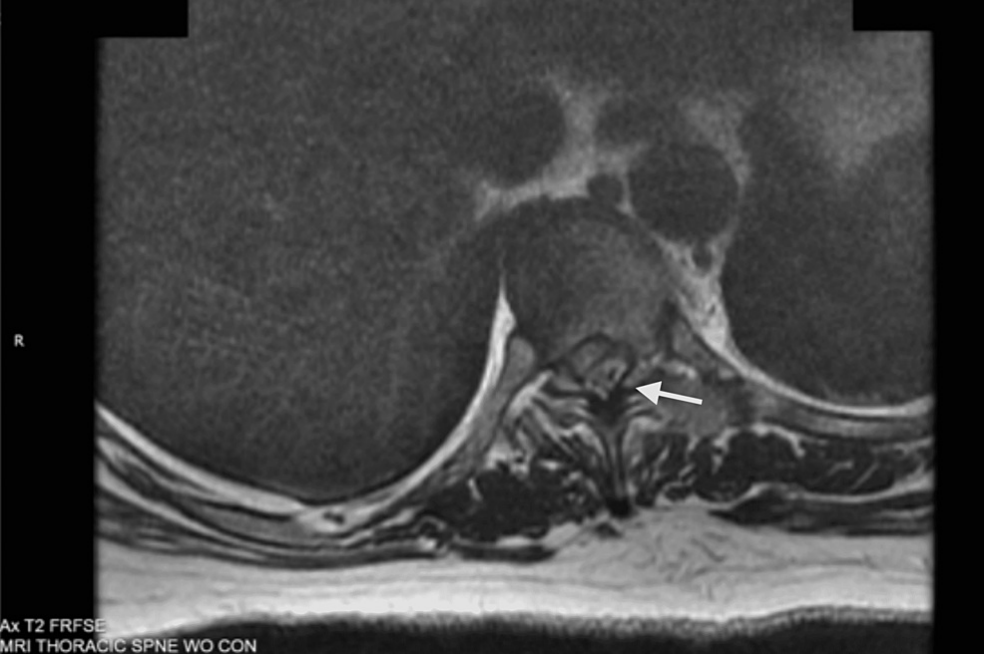
Axial view depicting dorsal epidural disc fragment centered at the T9-T10 level.

While the cervical spine MRI was unremarkable, her thoracic spine MRI revealed severe spinal cord compression and displacement at the T9-T10 level, secondary to a mixed-signal intensity soft tissue mass in the posterolateral spinal canal to the left of the midline (Figures 1, 2). In addition, T2 hyperintensity was noted within the compressed thoracic spinal cord, consistent with edema. Thoracic MRI was repeated with contrast and again showed a left dorsal epidural lesion centered at the T9-T10 level. Neurosurgery was again consulted for these findings, and they recommended transferring the patient to the intensive care unit and initiating 4 mg of IV dexamethasone every four hours in preparation for surgery.

The following morning, the patient underwent a T9-T10 laminectomy with excision of the lesion causing spinal cord compression. The intraoperative report indicated that the lesion was located beneath the ligamentum flavum on the left side and extended around the lateral aspect of the epidural space to the posterior longitudinal ligament. The lesion was noted to be encapsulated, and the frozen section at the time showed fibrous tissue without malignancy. Postoperative recommendations included ongoing management with oxycodone 10 mg every six hours as needed, baclofen 10 mg three times daily, and a dexamethasone taper of 4 mg three times daily for three days, followed by 4 mg twice daily for three days, 2 mg twice daily for three days, and 2 mg daily for three days.

Ultimately, the patient was admitted back to acute inpatient rehabilitation on postoperative day six. During this time, she made functional gains with a slight reduction in pain and spasms. She initially required maximum assistance from two therapists for transfers and bed mobility; however, by discharge, she required moderate assistance from one treating therapist. Likewise, on admission, she was unable to ambulate, but, by discharge, she could self-propel 150 feet using a wheelchair under supervision of the treating therapist. The pathology report of the spine mass specimen later revealed collagenous tissue with fibroblastic proliferation, consistent with a sequestered fragment of the herniated intervertebral disc. After her second acute inpatient rehabilitation course, she was discharged to a subacute rehabilitation facility. She was later discharged home and was able to ambulate independently with a walker.

## Discussion

GBS is a clinical diagnosis often supported by the laboratory finding of albuminocytological dissociation on CSF analysis and nerve conduction abnormalities in electrodiagnostic studies [3,4]. In our case, the patient initially presented with progressive bilateral lower extremity weakness and absent bilateral lower extremity deep tendon reflexes, which are classic signs of GBS. The finding of albuminocytological dissociation on CSF analysis further supported the diagnosis and drove initial management with dexamethasone followed by a five-day course of IVIG. Although the patient made initial functional improvements, new-onset bilateral upper extremity ulnar paresthesias prompted the clinician team to obtain further MRIs of the cervical and thoracic spine. This eventually led to the diagnosis of myelopathy due to a left dorsal epidural disc fragment at the T9-T10 level, which was definitively treated with T9-T10 laminectomy, followed by surgical excision of the sequestered mass.

Symptomatic disc herniations most commonly occur at the cervical and lumbar levels, with only 0.15% to 4% of all symptomatic herniations occurring at the level of the thoracic spine [5,6]. Migration of a thoracic herniated disc into the posterior epidural space is an even rarer occurrence. We could identify only nine other reported cases of a sequestered thoracic disc fragment localized to the posterior epidural space in the English-language literature [7-15]. Disc herniation typically occurs in the anterior epidural space by perforation of the annulus fibrosis and posterior longitudinal ligament, but movement further posteriorly is typically restricted by the meningovertebral ligaments and limited to sequestered disc fragments [15]. MRI is the diagnostic modality of choice and often displays a hypointense mass on T1-weighted images with signal hyperintensity on T2-weighted images, which is consistent with our case and other case studies [7-9,16]. Including our patient, eight of the ten cases presented with abrupt lower extremity motor weakness [7-15]. Deep tendon reflexes and the Babinski sign were inconsistently reported.

Because GBS can present similarly to other conditions, making an early definitive diagnosis can be challenging. Our patient initially presented with bilateral lower extremity motor weakness and absent deep tendon reflexes. While these findings are typical of the disease, GBS is a clinical diagnosis that requires the exclusion of other possible diagnoses as well as supportive testing. In our case, the diagnosis of GBS was driven by the supportive finding of albuminocytological dissociation on LP fluid analysis, which refers to the elevation of total CSF protein without pleocytosis. One review of 2,627 patients who underwent an LP found that the most common clinical diagnoses associated with true albuminocytological dissociation were polyneuropathy (21%), benign headaches (14%), seizures (9%), and intra-axial or extra-axial tumors (8%) [17]. Albuminocytological dissociation is commonly seen in inflammatory polyneuropathies due to disruption of the blood-nerve barrier; however, it is a non-specific indicator that can be associated with numerous pathologies. It is a misconception that albuminocytological dissociation confirms, rather than supports, the diagnosis of GBS, as only approximately 64% of patients exhibit the finding [4]. In our case, severe spinal cord compression likely disrupted CSF flow and led to the positive laboratory finding. Nerve conduction studies can also be used to support the diagnosis of GBS, but findings may be normal early in the disease course or in patients with atypical clinical variants [18]. Because nerve conduction abnormalities classically reach peak intensity more than two weeks after symptom onset, the utility of electrodiagnostic studies in diagnosis is limited but quite valuable when differentiating subtypes of GBS and determining prognosis [18]. Our patient did not have nerve conductions performed.

MRI is not routinely used in the diagnostic pathway of GBS but can be helpful for excluding other potential diagnoses, including spinal cord infection, inflammation, compression, and malignancy [1]. Nerve root enhancement of the cauda equina occurs in GBS, and one study of 24 patients reported prominent enhancement with gadolinium on lumbosacral MRI in 18 of 19 patients with classic GBS and two of five patients with variant GBS [19]. The authors further noted that lumbosacral MRI with gadolinium may be useful in cases with equivocal electrodiagnostic findings. One study of 494 adults diagnosed with GBS found that most patients exhibited tetraparesis and only 8% exhibited paraparesis, and all paraparetic patients exhibited decreased reflexes of the lower extremities [20]. Our patient also presented with paraparesis and absent deep tendon reflexes. It has been suggested that when GBS is the working diagnosis in a patient with paraparesis or an abnormal spinal sensory level, an MRI of the spinal cord should be performed to exclude spinal cord compression or transverse myelitis [4]. We agree with this statement and further suggest that in any patient in whom the diagnosis of GBS is uncertain, an MRI of the entire spine should be obtained to rule out other possible pathologies. In our case, failure to obtain thoracic imaging led to a significant delay in accurate diagnosis and definitive treatment, which may have adversely affected our patient’s ultimate functional outcome.

## Conclusions

Although this patient presented with progressive bilateral lower extremity weakness and absent bilateral lower extremity deep tendon reflexes, which are classic signs of GBS, she failed to improve despite standard treatment. Moreover, the finding of albuminocytological dissociation on CSF analysis supported and drove the clinical diagnosis of acute GBS. Dependency on positive albuminocytological dissociation as the most important diagnostic indicator in cases of potential GBS can lead to errors in diagnostic accuracy and delay treatment of the appropriate pathology. Furthermore, clinicians should remain mindful that GBS is a diagnosis of exclusion, and an MRI of the entire spine should be considered in any patient in whom the diagnosis of GBS is uncertain.
